# PEDOT: PSS/AuNPs-Based Composite as Voltammetric Sensor for the Detection of Pirimicarb

**DOI:** 10.3390/polym15030739

**Published:** 2023-01-31

**Authors:** Andrei E. Deller, Bruna M. Hryniewicz, Camila Pesqueira, Rayta Paim Horta, Bruno José Gonçalves da Silva, Saddam Weheabby, Ammar Al-Hamry, Olfa Kanoun, Marcio Vidotti

**Affiliations:** 1 Grupo de Pesquisa em Macromoléculas e Interfaces, Universidade Federal do Paraná (UFPR), Curitiba 81531-980, PR, Brazil; 2 Grupo de Cromatografia e Técnicas de Microextração, Departamento de Química, Universidade Federal do Paraná–UFPR, C.P. 19032, Curitiba 81531-980, PR, Brazil; 3 Measurement and Sensor Technology, Chemnitz University of Technology, 09126 Chemnitz, Germany

**Keywords:** pirimicarb, electrochemical sensor, modified electrode, conductive polymers, composites, PEDOT:PSS

## Abstract

An electrochemical sensor for the pesticide Pirimicarb (PMC) has been developed. A screen-printed electrode (SPCE) was used and modified with the conducting polymer poly (3,4-ethylenedioxythiophene) (PEDOT) and gold nanoparticles (AuNPs) to enhance electrochemical proprieties. Electrode characterizations were performed using scattering electron microscopy (SEM) and cyclic voltammetry (CV). With the SPCE/PEDOT:PSS/AuNPs modified electrode, a new peak at 1.0 V appeared in the presence of PMC related to the PMC oxidation. To elucidate the mechanism of PMC oxidation, Gas Chromatography-Mass Spectrometry (GC-MS), where two major peaks were identified, evidencing that the device can both detect and degrade PMC by an electro-oxidation process. Exploring this peak signal, it was possible the sensor development, performing detection from 93.81–750 µmol L^−1^, limits of quantification (LOQ) and detection (LOD) of 93.91 µmol L^−1^ and 28.34 µmol L^−1^, respectively. Thus, it was possible to study and optimization of PMC degradation, moreover, to perform detection at low concentrations and with good selectivity against different interferents using a low-cost printed electrode based on graphite modified with conductive polymer and AuNPs.

## 1. Introduction

Electrochemistry can perform an important role in the monitoring and remediation of environmental contaminants, which have been used in the development of electrochemical sensors to quantify the levels of these contaminants and electrocatalytic systems for the degradation of these substances [1,2,3]. In addition, the electrocatalytic signal generated can be explored for sensing performance using some electrochemical techniques such as voltammetry or amperometry. Even if a molecule does not show an electrocatalytic signal, it does not mean that it cannot be quantified because it is possible to explore another signal, such as adsorption, blocking effect, etc., using a technique such as electrochemical impedance spectroscopy (EIS), that is very sensitive to small perturbations in the electrochemical system [3,4].

Electrocatalysis allows converting a molecule into a less harmful product to the environment in an optimized process, so it is possible to enhance the kinetics, efficiency, and selectivity of the conversion. A good electrocatalyst should promote a low overpotential (η), reducing the energy required for the reaction to occur. For the oxidation process, the reaction occurs in a less positive potential, and for reductions in a less negative potential. [2,3]. This decrease in η value is particularly difficult for some pesticides due to their high redox potentials. For example, some carbamates may have high degradation potentials and, in addition, may have complex degradation mechanisms [5,6].

**Figure 1:**
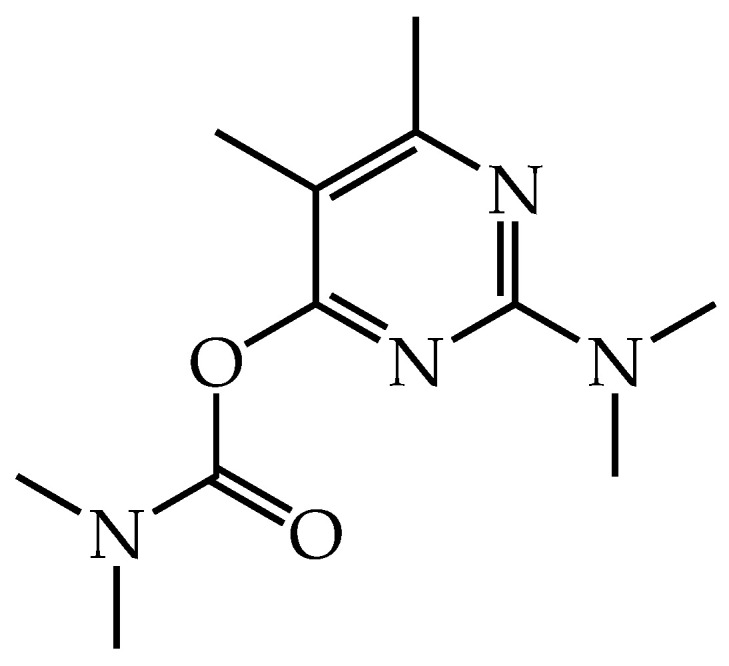
Chemical structure of pirimicarb.

Among the pesticides of this class, Pirimicarb (PMC) ([2-(dimethylamino)-5,6-dimethylpyrimidin-4-yl] N, N-dimethyl carbamate) (Figure 1) was classified by the United States Environmental Protection Agency (U.S. EPA) as potentially carcinogenic to humans (II-III class). In addition, the World Health Organization (WHO) has classified PMC as moderately dangerous [7], and like other N-carbamate pesticides, the toxicity is related to the inhibition of acetylcholinesterase (AChE) at synapses in the brain and neuromuscular junctions in skeletal muscles; this inhibition causes overstimulation of electrical activity and can accumulate acetylcholine (Ach) at toxic levels [5]. The publications that perform the detection of PMC mainly use techniques of liquid chromatography [8,9,10,11], gas chromatography [12,13], biosensors [14,15], polarography [16], and quartz crystal microbalance [17]. As far as we know, only one publication reports an electrochemical sensor to detect PMC [18]. 

The small number of electrochemical studies may be due to the difficulty in oxidizing PMC where the oxidation happens in high potentials near water electrolysis potential. Combining improved electrode materials with electrochemical techniques can generate devices that perform sensing of carbamate pesticides such as PMC [19]. The advantage is that electrochemistry enables fast analysis, good portability, and high sensitivity against low concentrations of analytes [20,21,22]. Amongst the different electrode modifiers, conducting polymers stand out for applications in sensors due to their high conductivity and electroactivity, fast charge-transfer rates, and stability [23]. Poly (3,4-ethylenedioxythiophene) (PEDOT) is a promising conducting polymer for PMC detection since it has a large operating voltage window, and it has already been used for the detection of other pesticides and toxic organic molecules [24,25]. In addition, the decoration of PEDOT structures with gold nanoparticles (AuNPs) can enhance the electrocatalytic properties of the material and facilitate the PMC oxidation at the electrode surface [24].

Thus, new electrode materials must be produced to develop better methodologies for the study of PMC detection and degradation in addition to developing methodologies to quantify its levels in the environment in the future. In this context, this work aims to study the electrocatalytic oxidation of PMC, using a low-cost electrode material based on screen-printed carbon electrodes modified with PEDOT and AuNPs for its sensitive quantification.

## 2. Materials and Methods

### 2.1. Chemicals and Materials

All the solutions were prepared using ultrapure water (ElgaLab water 18 MΩ cm^−1^), gold chloride trihydrate (III) (HAuCl_4_.3H_2_O), ethylenediaminetetraacetic acid (EDTA), potassium chloride (KCl), and Pirimicarb (PMC) which were all purchased from Aldrich. PEDOT:PSS was purchased from Heareus (1.3 wt% in H_2_O). Malathion, chlorpyrifos, aldicarb, glucose, ascorbic acid and dopamine were purchased from Thermo Fischer GmbH (Kandel, Germany).

The synthesis and characterization steps were performed in a Palmsens4 potentiostat (PalmSens B.V., Houten, The Netherlands) with a three-electrode electrochemical cell. Screen-printed carbon electrodes (SPCE, ItalSens IS-C) were purchased from PalmSens B.V., containing a graphite working electrode, a carbon counter electrode and a silver pseudoreference electrode. UV-Vis spectra were collected in a Cary60 spectrophotometer (Agilent Technologies, Santa Clara, CA, USA). Scanning electron microscopy (SEM) images were acquired in a TESCAN MIRA3 equipment with a SDD of 80 mm^2^ EDS detector.

### 2.2. Deposition and Characterization of the Composite Electrodes

The electrostatic deposition of PEDOT:PSS on SPCE was performed in an aqueous dispersion of 0.0013 wt% PEDOT:PSS, under a potential application of 1.25 V for 500 s. The AuNPs electrochemical synthesis on PEDOT:PSS modified electrode was conducted in a solution of 1.0 mmol L^−1^ HAuCl_4._3H_2_O, 0.17 mol L^−1^ K_2_HPO_4_, 36 mmol L^−1^ Na_2_SO_3_, and 0.48 mmol L^−1^ EDTA [26] by direct reduction of the gold salt, by applying -1.1 V with charge control of 300 mC cm^−2^. 

The electrochemical characterization of 500 µmol L^−1^ PMC by PEDOT:PSS and PEDOT:PSS/AuNPs modified electrodes was performed by cyclic voltammetry (CV) and the detection of different concentrations of PMC was conducted by linear sweep voltammetry (LSV) in acetate buffer pH 5.0 at 20 mV s^−1^.

### 2.3. PMC Oxidation and Detection

The interference study was performed by LSV in acetate buffer pH 5.0 at 20 mV s^−1^ in the presence of 250 µmol L^−1^ PMC and 250 µmol L^−1^ of each interferent. Chlorpyrifos was previously diluted in acetone due to its low solubility in water.

A 30 µL of 100 µmol L^−1^ PMC solution in acetate buffer pH 5.0 was electrolyzed by PEDOT:PSS/AuNPs for 2 h with the application of 1.1 V. The aliquot was collected by the end of the electrolysis and the products were extracted with ethyl acetate. The aliquot was dried and redispersed in 1 mL ethyl acetate before gas chromatography-mass spectrometry (GC-MS) analysis. The GC-MS analysis was conducted on a Shimadzu system consisting of a GC2010 Plus gas chromatograph hyphenated to a TQ8040 triple quadrupole mass spectrometer (Shimadzu, Kyoto, Japan). The GC was equipped with an AOC5000 auto-injector and a split/splitless injector. The chromatographic separation occurred in a GC column SH-RTX-5 ms (30 m × 0.25 mm × 0.25 µm) with helium 5.0 as the carrier gas at a flow rate of 1.0 mL min^−1^. A sample volume of 1 µL was injected in a split ratio of 1:10. Both the injector and ion source temperatures were set at 250 °C. The interface temperature was 275 °C. The GC oven temperature was kept at 50 °C for 0.5 min, followed by 15 °C/min to 300 °C (2.83 min). The total analysis time was 20 min. The triple quadrupole mass spectrometer was operated in full scan (m/z 50–350) with electron ionization at 70 eV. 

## 3. Results and Discussion

### 3.1. Microscopic and Spectroscopic Characterization of the Composite Electrodes

**Figure 2:**
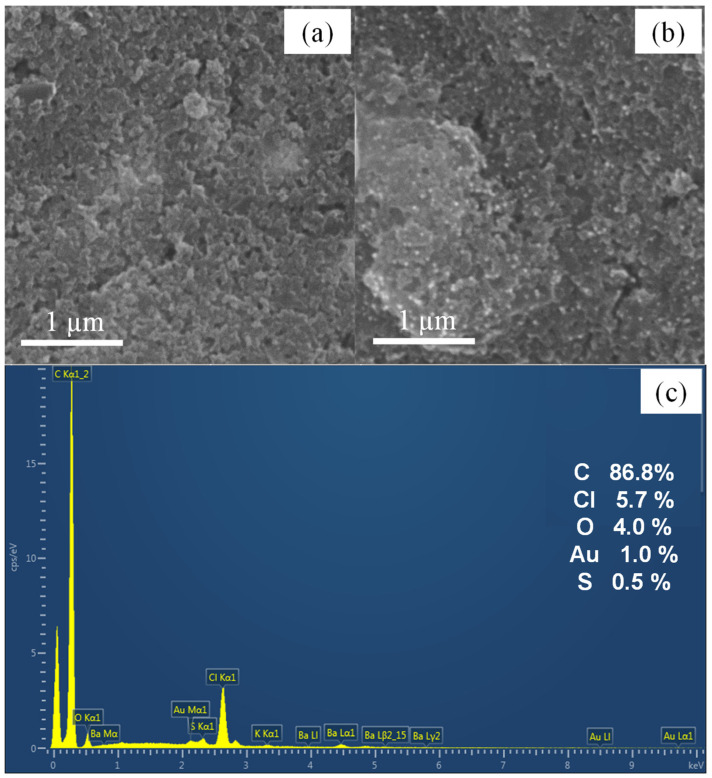
Representative secondary electron SEM images were obtained from (**a**) PEDOT:PSS and (**b**) PEDOT:PSS/AuNPs modified electrode. Magnification = 40 kx, voltage = 15 kV. (**c**) EDS spectra of PEDOT:PSS/AuNPs modified electrode.

PEDOT:PSS and PEDOT:PSS/AuNPs modified electrodes were characterized by SEM images in secondary electron mode, using a voltage of 15 kV and a magnification of 40 kx, as shown in Figure 2a,b, respectively. The image indicates that PEDOT is predominantly deposited as a thin film (Figure 2a), with the roughness probably due to the porous structure of the graphite (Figure S1), differently than other substrates, such as platinum or ITO where a globular morphology is found [3,4]. The presence of AuNPs can be verified in Figure 2b, where the nanoparticles are readily observed all alongside the PEDOT surface and confirmed by EDX spectra (Figure 2c). 

### 3.2. Voltammetric and Chromatography Studies of PMC Oxidation 

**Figure 3:**
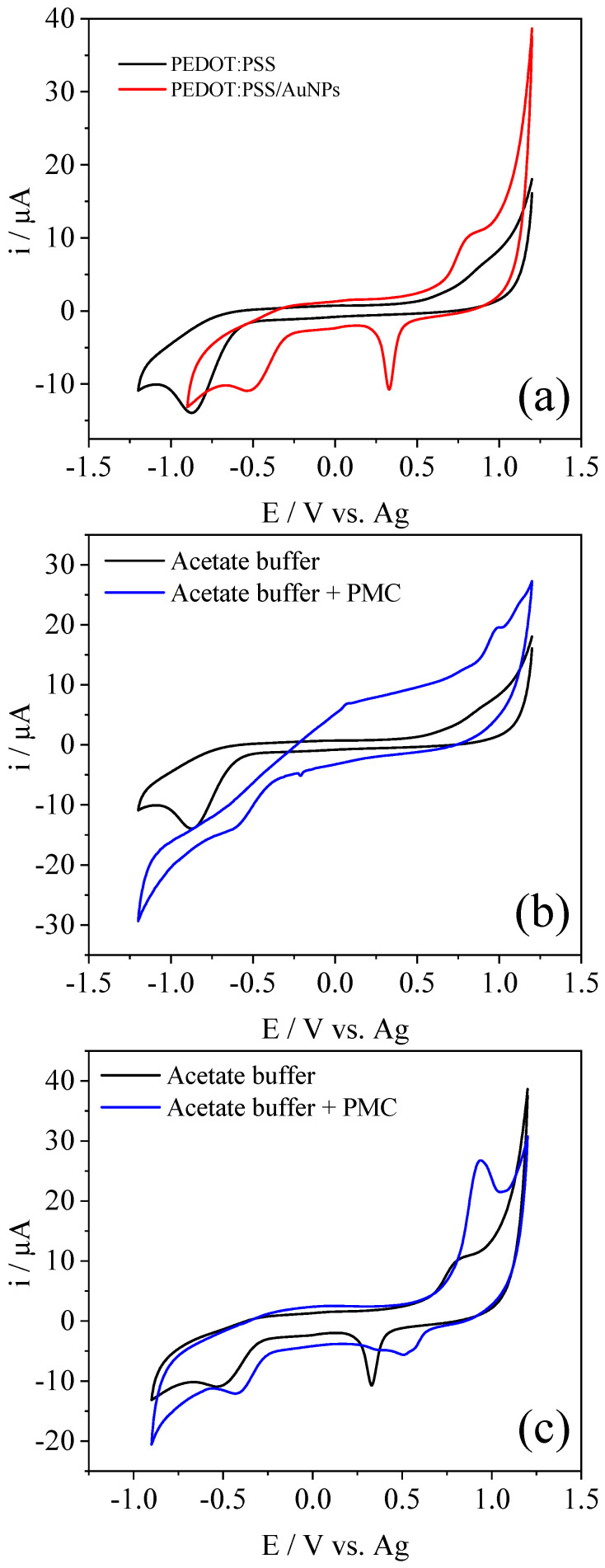
(**a**) CV at 20 mV s^−1^ of PEDOT:PSS and PEDOT:PSS/AuNPs SPCE modified electrodes in acetate buffer. CVs of (**b**) PEDOT:PSS and (**c**) PEDOT:PSS/AuNPs in the absence and the presence of 500 µmol L^−1^ PMC at 20 mV s^−1^. Electrolyte = Acetate buffer pH 5.0.

PEDOT:PSS and PEDOT:PSS/AuNPs modified electrodes were characterized by CV in acetate buffer pH 5, as shown in Figure 3a. The voltammogram of PEDOT:PSS shows the characteristic PEDOT redox processes with a sharp reduction peak near −0.9 V, with a possible contribution from the oxygen reduction reaction process and a broad oxidation peak at 0.9 V. In the presence of AuNPs, PEDOT processes shifted towards less energetic potentials, −0.52 V and 0.8 V, respectively. The charge transfer and intercalation processes of the ions inside the polymeric matrix to maintain the charge neutrality upon the redox reactions were facilitated in the presence of the nanoparticles. This behavior was observed for PEDOT and gold nanoparticle composites [4]. In addition, a new reduction peak at +0.33 V appeared, related to the reduction of gold oxides formed in the forward scan [27].

The materials were characterized in the presence of 500 µmol L^−1^ PMC, as shown in Figure 3b,c for PEDOT:PSS and PEDOT:PSS/AuNPs, respectively. For PEDOT:PSS, the presence of the pesticide led to a drastic change in the voltammetric profile, probably due to the strong adsorption of this compound with the PEDOT:PSS structure, making the charge transfer processes difficult. However, a new peak at 1.0 V appeared in the presence of PMC, which is related to the PMC oxidation. In the literature, the mechanism of PMC oxidation is described by four processes [18]. In the first process, the N-H in the pyrimidine ring is oxidized; in the second, the oxidation of the tertiary amine occurs; in the third occurs the oxidation of the other amine in the pyrimidine group; and in the last occurs the formation of hydroxyl radicals due to water oxidation, forming the final degradation products. In the voltammogram of PEDOT:PSS/AuNPs, the presence of PMC slightly changed the PEDOT:PSS redox processes, while an intense oxidation peak at 0.94 V appeared, indicating that the PMC can be detected by this material, as shown in Figure 3c. In the CV at 40 mV s^−1^, a second PMC oxidation peak can be visualized (Figure S2). As studied by Paixão et. al, the second oxidation process is controlled by the adsorption of the species on the electrode surface in scan rates up to 100 mV s^−1^. It is possible that in the voltammogram at 20 mV s^−1^, both oxidation processes take place in the same potential (approximately 0.94 V), and at 40 mV s^−1^, the adsorption of the intermediates formed in the first oxidation process is limited by the scan rate and an overpotential appears for their oxidation [18]. The other oxidation processes could not be seen in the voltammograms due to the window voltage limitation. CVs of the PEDOT:PSS/AuNPs that was cleaned with a copious amount of acetate buffer after the PMC detection is shown in Figure S3. It can be seen that the PMC detection led only to a small change in the PEDOT:PSS redox processes and a huge decrease in the AuNPs reduction peak, indicating that the PMC is mainly adsorbed in the metallic nanoparticles.

**Figure 4:**
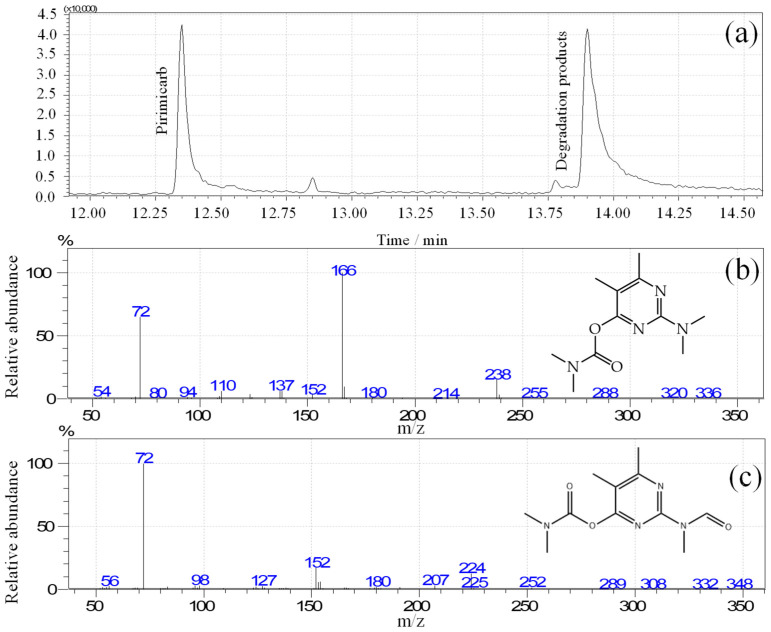
(**a**) Chromatogram of the PMC solution after the electrochemical oxidation by PEDOT:PSS/AuNPs modified electrode. Mass spectra of (**b**) pirimicarb and (**c**) degradation product carbamic acid, dimethyl-, 2-(formylmethylamino)-5,6-dimethyl-4-pyrimidinyl ester.

To better understand the mechanism of PMC oxidation, an aliquot of acetate buffer and 100 µmol L^−1^ PMC was submitted to chronoamperometry by PEDOT:PSS/AuNPs at 1.1 V for 2 h. The aliquot was collected and analyzed by injection of 1 µL in GC-MS, and the chromatogram is shown in Figure 4a. Based on the Nist05 mass spectrum library database (GCMSsolution^®^ version 4.20), two major peaks were identified, with at least 86% of similarity. The first one (retention time of 12.857 min) with m/z ratios of 72, 166, and 238 (Figure 4b), related to the PMC that was not completely consumed during the reaction, and the second one (retention time of 13.850 min) with m/z ratios of 72, 153, and 224 (Figure 4c), which is described as a degradation product of the reaction. These results confirm that PMC is degraded by hydroxyl radicals due to water oxidation [18], showing that the sensor can both detect and degrade PMC by an electro-oxidation process.

### 3.3. PMC Detection and Interference Study

**Figure 5:**
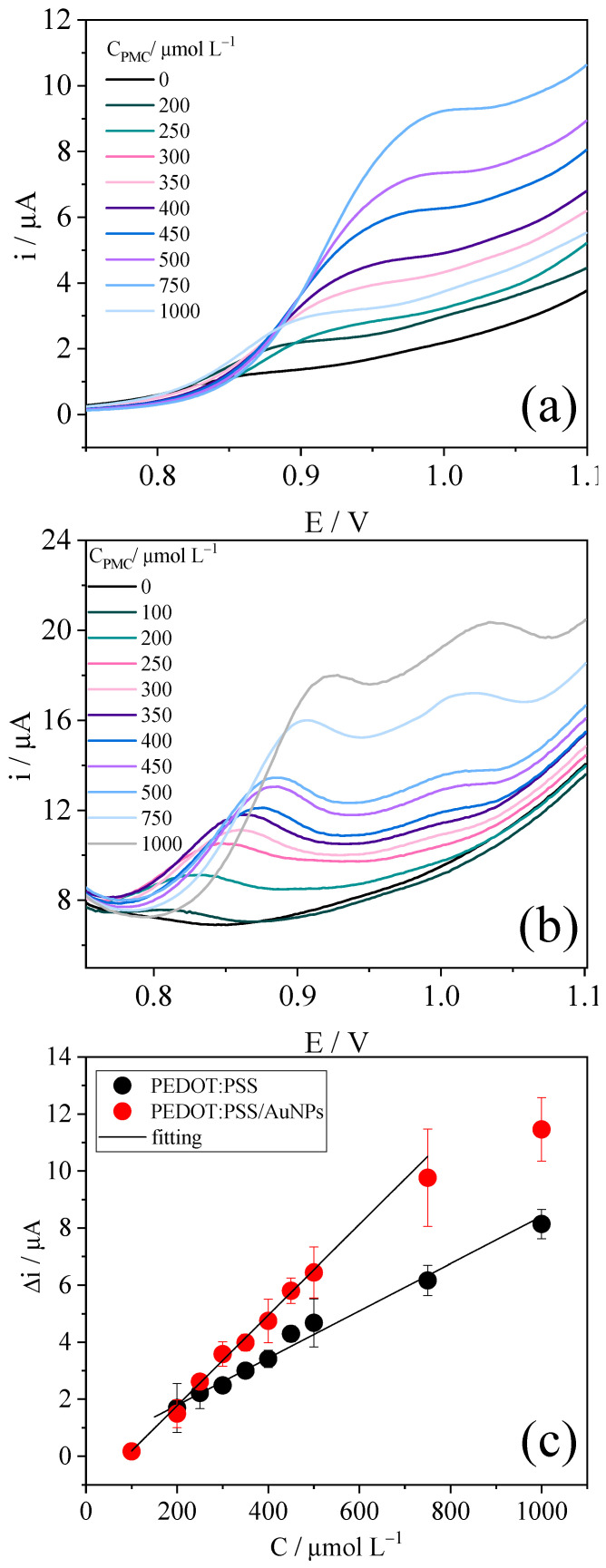
LSV response of (**a**) PEDOT:PSS and (**b**) PEDOT:PSS/AuNPs in the presence of different PMC concentrations in acetate buffer pH 5. Scan rate = 20 mV s^−1^. The (**c**) analytical curves for PMC detection in triplicate.

The detection of different PMC concentrations was performed using linear voltammetry between 0.75 and 1.1 V. The results were obtained in triplicate, and one of the replicates is shown in Figure 5a,b, for PEDOT:PSS and PEDOT:PSS/AuNPs, respectively. For PEDOT:PSS, only one PMC peak was visible in the liner voltammogram at 0.9 V, which was shifted to 0.98 V with increasing concentrations, probably due to the adsorption of PMC on the material surface, hindering the electron transfer process. For PEDOT:PSS/AuNPs, two oxidation peaks appeared. The first one, initially at 0.81 V, appeared in the smaller concentrations, while the second one, initially at 0.97 V, became evident in the highest concentrations. The first and second peaks shifted to 0.92 and 1.03 V with increasing concentrations, respectively. The analytical curves were constructed using the current value of the single peak for PEDOT:PSS electrode and the current value of the first peak for PEDOT:PSS/AuNPs electrode. The results are shown in Figure 5c. It can be seen a linear correlation between the increase in PMC concentration and the peak oxidation increase, proving that both electrodes can perform PMC detection. The limit of detection (LOD = 3σ/S) and limit of quantification (LOQ = 10σ/S) were calculated using the standard deviation (σ) of ten LSV measurements in the absence of PMC and the sensitivity (S) obtained by the analytical curve as shown in Figure 5c. The linear range for PMC detection using PEDOT:PSS was 227.71 to 1000.00 µmol L^−1^, while for PEDOT:PSS/AuNPs it was 93.81 to 750.00 µmol L^−1^. Table 1 shows the analytical parameters obtained for both materials. PEDOT:PSS/AuNPs showed a smaller LOD and LOQ and a greater sensitivity for PMC detection, evidencing the potentiality of using this material for future environmental protection systems. 

There is only one electrochemical sensor in the literature for PMC detection. Although the work from Paixão et al. [18] showed better analytical performance, with a LOD of 1.24 µmol L^−1^ and a sensitivity of 90.2 mA L mol^−1^, the authors used a boron-doped diamond (BDD) electrode for the detection [18]. This is an ideal system with a very low background signal, an extremely large potential window (>2 V), and it is very expensive and difficult to be produced. Herein, we could detect PMC using a disposable screen-printed electrode and a conducting polymer, which is a simpler and lower-cost platform and still obtain LOD and LOQ in the same order of magnitude. In this way, PEDOT:PSS/AuNPs can be seen as a good substitute for BDD in the detection of PMC.

To verify the selectivity of the material, different interferents were tested together with PMC solutions. Tests using linear voltammetry were performed first in the presence of 250 µmol L^−1^ PMC only, and then with PMC at the same concentration in the presence of 250 µmol L^−1^ of each interferent. The results are shown in Figure S4. The effect of the interferent was calculated by the variation in the peak current value in relation to the peak current for the PMC. As shown in Table 2, only chlorpyrifos and malathion had a significative interference in the signal (bigger than 5%). However, the interference is still low (<11%). Other substances tested did not interfere significantly with the PMC signal. Especially, Aldicarb has shown only 3.72% interference, a small value considering that it is also a carbamate just like PMC. Therefore, the proposed sensor has good selectivity to detect PMC.

## 4. Conclusions

PMC is a carbamate pesticide difficult to detect using electrochemistry due to its high oxidation potential. However, in this research, we proposed a new electrochemical sensor to quantify PMC based on modified screen-printed carbon electrodes, which are inexpensive, portable, and easy to use. Besides that, modifying the surface with PEDOT and gold nanoparticles enhanced the electrochemical properties, making possible the oxidation of PMC in a less positive potential. The product of PMC oxidation was investigated by GC-MS experiments. Exploring the electrocatalytic effect made it possible to correlate the oxidation peak with the pesticide concentration, obtaining an electrochemical sensor to quantify PMC that is sensitive, selective, low-cost, and with a promising application for future environmental monitoring. 

## Figures and Tables

**Table 1:** Analytical performance of PEDOT:PSS and PEDOT:PSS/AuNPs for PMC detection.

Electrode	Sensitivity/ mA L mol^−1^	LOD/ µmol L^−1^	LOQ/ µmol L^−1^	Linear Range/ µmol L^−1^	R^2^
PEDOT:PSS	8.27	68.31	227.71	227.71–1000.00	0.9778
PEDOT:PSS /AuNPs	15.89	28.34	93.81	93.81–750.00	0.9974

**Table 2:** Tested interferents and their respective interferences in the voltammetric signal.

Interferences	±%
Chlorpyrifos	7.63
Aldicarb	3.72
Malathion	10.95
Glucose	3.29
Ascorbic Acid	0.32
Dopamine	0.35

## Data Availability

No new data were created or analyzed in this study. Data sharing is not applicable to this article.

## Supplementary Materials

The following supporting information can be downloaded at https://www.mdpi.com/article/10.3390/polym15030739/s1: Figure S1. SEM image of bare SPCE.; Figure S2. Cyclic voltammograms of PEDOT:PSS/AuNPs in acetate buffer pH 5 (a) without PMC (b) and in the presence of 500 µmol L^−1^ PMC at 40 mV s^−1^, Figure S3. Cyclic voltammograms of PEDOT:PSS/AuNPs in acetate buffer pH 5 (a) before and (b) after the detection of 500 µmol L^−1^ PMC, Figure S4. Linear voltammograms of 250 µmol L^−1^ PMC in the presence of different interferents at the same concentration.
