# Supplementary material for: PEDOT: PSS/AuNPs-Based Composite as Voltammetric Sensor for the Detection of Pirimicarb
Source: Polymers (Basel). 2023 Jan 31;15(3):739. doi: 10.3390/polym15030739 (PMC9919558; doi:10.3390/polym15030739)
Supplement: Supplementary file 1 [file polymers-15-00739-s001.zip › polymers-2139136-supplementary.pdf]

Supplementary material for

PEDOT: PSS/AuNPs-Based Composite as Voltammetric Sensor for the Detection of Pirimicarb

Andrei E. Deller [1,†], Bruna M. Hryniewicz [1,†], Camila Pesqueira [1], Rayta Paim Horta [2], Bruno José Gonçalves da Silva [2], Saddam Weheabby [3,*], Ammar Al-Hamry [3], Olfa Kanoun [3] and Marcio Vidotti [1,*]

[1] Grupo de Pesquisa em Macromoléculas e Interfaces, Universidade Federal do Paraná (UFPR), Curitiba 81531-980, PR, Brazil
[2] Grupo de Cromatografia e Técnicas de Microextração, Departamento de Química, Universidade Federal do Paraná–UFPR, C.P. 19032, Curitiba 81531-980, PR, Brazil
[3] Measurement and Sensor Technology, Chemnitz University of Technology, 09126 Chemnitz, Germany;
* Correspondence: mvidotti@ufpr.br
† These authors contributed equally to this work.

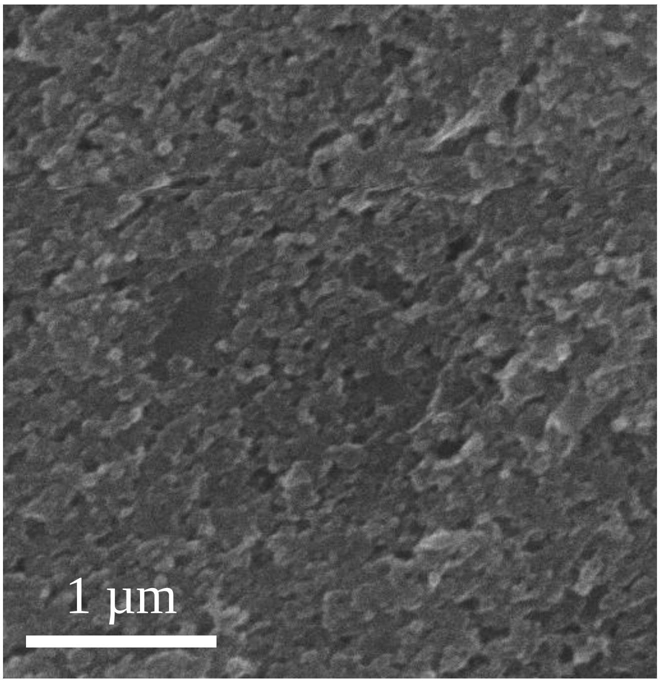

Figure S1. SEM image of bare SPCE.

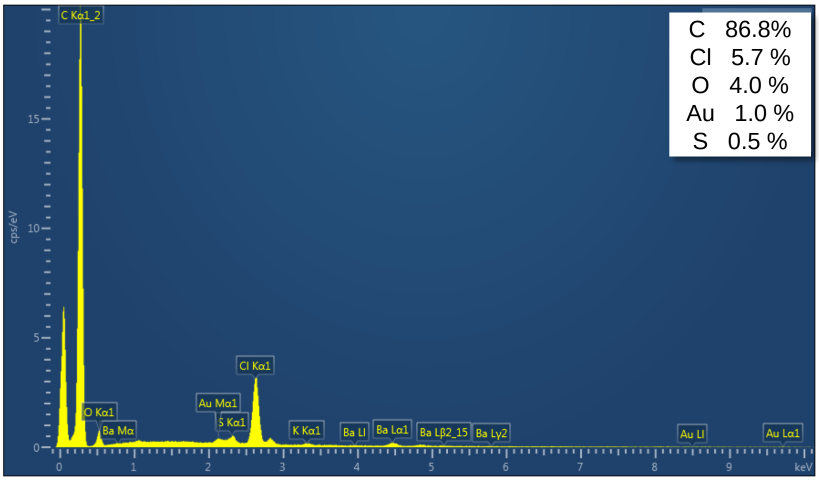

Figure S2. EDS spectra of PEDOT:PSS/AuNPs modified SPCE.

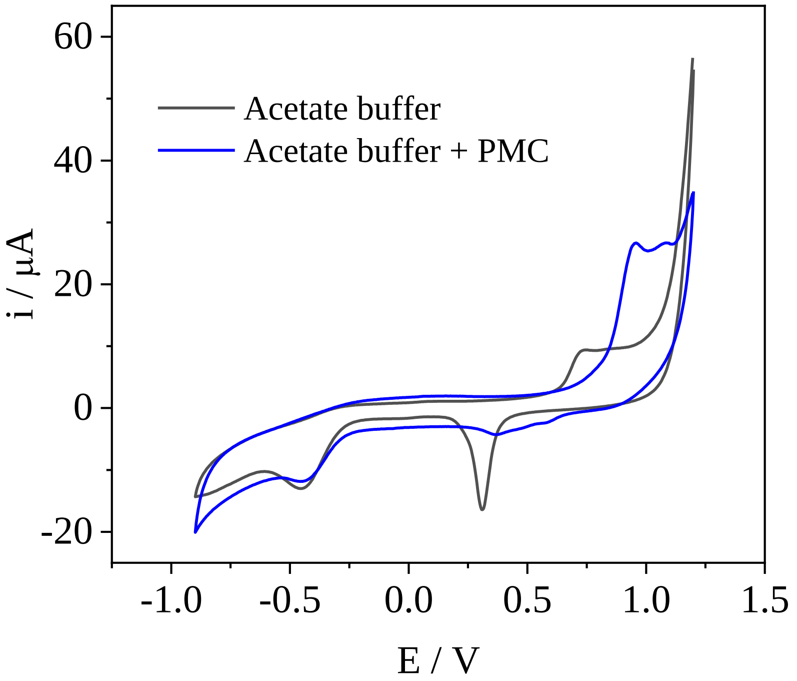

Figure S3. Cyclic voltammograms of PEDOT:PSS/AuNPs in acetate buffer pH 5 (**a**) without PMC (**b**) and in the presence of 500µmol L^{-1} PMC at 40 mV s^{-1}.

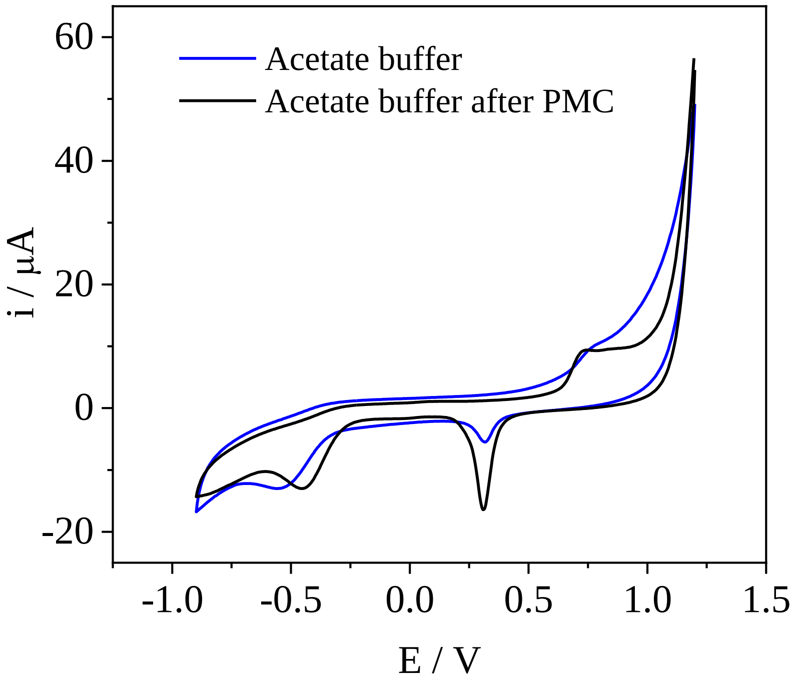

Figure S4. Cyclic voltammograms of PEDOT:PSS/AuNPs in acetate buffer pH 5 (**a**) before and (**b**) after the detection of 500 µmol L^{-1} PMC.

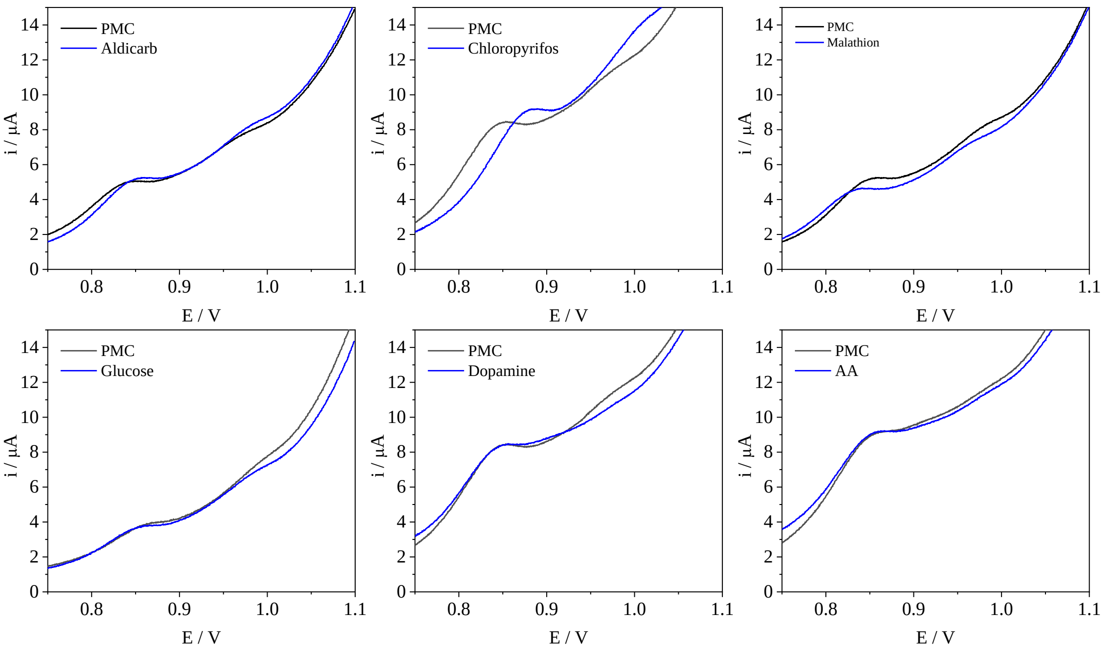

Figure S5. Linear voltammograms of 250 μmol L^{-1} PMC in the presence of different interferents at the same concentration.
